# Supplementary material for: Molecular phylogenies confirm the presence of two cryptic Hemimycale species in the Mediterranean and reveal the polyphyly of the genera Crella and Hemimycale (Demospongiae: Poecilosclerida)
Source: PeerJ. 2017 Mar 7;5:e2958. doi: 10.7717/peerj.2958 (PMC5344016; doi:10.7717/peerj.2958)
Supplement: Supplemental Information 1 — 18S, 28S, and COI partitions used in the phylogenetic study and deposited at GenBank. [file peerj-05-2958-s001.rtf]

>Seq1_C.cyathophora_ind1_RS [organism=Crella cyathophora] Individual1 Red SeaCTTCGCCAGGATCAATGTTGGGGGACGATCATTTGTATAATGTAATTGTGACTGCACACGCTTTCGTAATGATTTTTTTTTTAGTTATGCCAGTAATGATTGGAGGGTTTGGAAATTGATTAGTGCCCTTATATATTGGGGCGCCCGATATGGCTTTTCCTAGATTAAATAATATAAGTTTTTGATTATTACCTCCGGCCTTAAGTTTGTTGTTAGCATCTGCTTTCGTGGAACAAGGGGCAGGGACAGGGTGAACTGTTTATCCTCCTTTATCAGGAAATCAGGCCCATTCTGGGGGATCGGTGGATTTAGTAATTTTCAGCCTACATTTAGCGGGAATTTCTTCAATTTTAGGTGCTATGAATTTTATTACTACAATAATAAATATGCGGGTGACAGGCATGACTTTTGACAGATTGCCTTTATTTGTCTGATCTATTTTAGTTACTGCTGTTTTATTGTTGTTGTCTTTACCTGTATTAGCAGGGGCGATTACTATGCTGTTAACGGATAGAAATTTTAATACTGCTTTTTT>Seq2_C.cyathophora_ind2_RS [organism=Crella cyathophora] Individual2 Red SeaCTTCGCCAGGATCAATGTTGGGGGACGATCATTTGTATAATGTAATTGTGACTGCACACGCTTTCGTAATGATTTTTTTTTTAGTTATGCCAGTAATGATTGGAGGGTTTGGAAATTGATTAGTGCCCTTATATATTGGGGCGCCCGATATGGCTTTTCCTAGATTAAATAATATAAGTTTTTGATTATTACCTCCGGCCTTAAGTTTGTTGTTAGCATCTGCTTTCGTGGAACAAGGGGCAGGGACAGGGTGAACTGTTTATCCTCCTTTATCAGGAAATCAGGCCCATTCTGGGGGATCGGTGGATTTAGTAATTTTCAGCCTACATTTAGCGGGAATTTCTTCAATTTTAGGTGCTATGAATTTTATTACTACAATAATAAATATGCGGGTGACAGGCATGACTTTTGACAGATTGCCTTTATTTGTCTGATCTATTTTAGTTACTGCTGTTTTATTGTTGTTGTCTTTACCTGTATTAGCAGGGGCGATTACTATGCTGTTAACGGATAGAAATTTTAATACTGCTTTTTT>Seq3_C.cyathophora_ind3_IND [organism=Crella cyathophora] Individual3 Indopacific OceanCTTCGCCAGGATCAATGTTGGGGGACGATCATTTGTATAATGTAATTGTGACTGCACACGCTTTCGTAATGATTTTTTTTTTAGTTATGCCAGTAATGATTGGAGGGTTTGGAAATTGATTAGTGCCCTTATATATTGGGGCGCCCGATATGGCTTTTCCTAGATTAAATAATATAAGTTTTTGATTATTACCTCCGGCCTTAAGTTTGTTGTTAGCATCTGCTTTCGTGGAACAAGGGGCAGGGACAGGGTGAACTGTTTATCCTCCTTTATCAGGAAATCAGGCCCATTCTGGGGGATCGGTGGATTTAGTAATTTTCAGCCTACATTTAGCGGGAATTTCTTCAATTTTAGGTGCTATGAATTTTATTACTACAATAATAAATATGCGGGTGACAGGCATGACTTTTGACAGATTGCCTTTATTTGTTTGATCTATTTTAGTTACTGCTGTTTTATTGTTGTTGTCTTTACCTGTATTAGCAGGGGCGATTACTATGCTGTTAACGGATAGAAATTTTAATACTGCTTTTTT>Seq4_C.cyathophora_ind4_IND [organism=Crella cyathophora] Individual4 Indopacific OceanCTTCGCCAGGATCAATGTTGGGGGACGATCATTTGTATAATGTAATTGTGACTGCACACGCTTTCGTAATGATTTTTTTTTTAGTTATGCCAGTAATGATTGGAGGGTTTGGAAATTGATTAGTGCCCTTATATATTGGGGCGCCCGATATGGCTTTTCCTAGATTAAATAATATAAGTTTTTGATTATTACCTCCGGCCTTAAGTTTGTTGTTAGCATCTGCTTTCGTGGAACAAGGGGCAGGGACAGGGTGAACTGTTTATCCTCCTTTATCAGGAAATCAGGCCCATTCTGGGGGATCGGTGGATTTAGTAATTTTCAGCCTACATTTAGCGGGAATTTCTTCAATTTTAGGTGCTATGAATTTTATTACTACAATAATAAATATGCGGGTGACAGGCATGACTTTTGACAGATTGCCTTTATTTGTTTGATCTATTTTAGTTACTGCTGTTTTATTGTTGTTGTCTTTACCTGTATTAGCAGGGGCGATTACTATGCTGTTAACGGATAGAAATTTTAATACTGCTTTTTT>Seq5_H.arabica_ind1_RS [organism=Hemimycale arabica] Individual1 Red SeaCTTCGCCAGGATCAATGTTGGGGGACGATCATTTGTATAATGTAATTGTGACTGCACACGCTTTCGTAATGATTTTTTTTTTAGTTATGCCAGTAATGATTGGAGGGTTTGGAAATTGATTAGTGCCCTTATATATTGGGGCGCCCGATATGGCTTTTCCTAGATTAAATAATATAAGTTTTTGATTATTACCTCCGGCCTTAAGTTTGTTGTTAGCATCTGCTTTCGTGGAACAAGGGGCAGGGACAGGGTGAACTGTTTATCCTCCTTTATCAGGAAATCAGGCCCATTCTGGGGGATCGGTGGATTTAGTAATTTTCAGCCTACATTTAGCGGGAATTTCTTCAATTTTAGGTGCTATGAATTTTATTACTACAATAATAAATATGCGGGTGACAGGCATGACTTTTGACAGATTGCCTTTATTTGTCTGATCTATTTTAGTTACTGCTGTTTTATTGTTGTTGTCTTTACCTGTATTAGCAGGGGCGATTACTATGCTGTTAACGGATAGAAATTTTAATACTGCTTTTTT>Seq6_H.arabica_ind2_RS [organism=Hemimycale arabica] Individual2 Red SeaCTTCGCCAGGATCAATGTTGGGGGACGATCATTTGTATAATGTAATTGTGACTGCACACGCTTTCGTAATGATTTTTTTTTTAGTTATGCCAGTAATGATTGGAGGGTTTGGAAATTGATTAGTGCCCTTATATATTGGGGCGCCCGATATGGCTTTTCCTAGATTAAATAATATAAGTTTTTGATTATTACCTCCGGCCTTAAGTTTGTTGTTAGCATCTGCTTTCGTGGAACAAGGGGCAGGGACAGGGTGAACTGTTTATCCTCCTTTATCAGGAAATCAGGCCCATTCTGGGGGATCGGTGGATTTAGTAATTTTCAGCCTACATTTAGCGGGAATTTCTTCAATTTTAGGTGCTATGAATTTTATTACTACAATAATAAATATGCGGGTGACAGGCATGACTTTTGACAGATTGCCTTTATTTGTCTGATCTATTTTAGTTACTGCTGTTTTATTGTTGTTGTCTTTACCTGTATTAGCAGGGGCGATTACTATGCTGTTAACGGATAGAAATTTTAATACTGCTTTTTT>Seq7_H.columella_ind3_Are [organism=Hemimycale columella] Individual3 Western Mediterranean Sea (Arenys)CTTCGCCAGGATCAATGCTAGGGGACGATCATTTGTATAACGTTCTTGTAACTGCGCATGCTTTTGTAATGATATTTTTTTTAGTTATGCCAGTAATGATTGGAGGGTTTGGTAACTGACTGGTGCCCTTGTATATTGGAGCGCCTGATATGGCTTTTCCTCGATTAAATAATATAAGTTTTTGATTATTGCCCCCGGCTTTAAGTTTATTGTTGGCGTCTGCTTTCGTGGAGCAAGGGGCAGGAACCGGATGAACTGTTTATCCCCCTCTCTCCGGGAATCAGGCTCATTCTGGCGGGTCAGTTGATTTAGTAATCTTTAGCCTACATTTAGCAGGAATTTCTTCAATTTTAGGCGCTATGAATTTTATTACTACAATAGTAAACATGCGGATAACGGGGATGACTTTTGATAGGTTGCCTTTATTTGTTTGATCTATTTTAGTTACCGCGGTTTTATTGTTATTGTCCTTACCTGTATTAGCGGGGGCTATTACCATGTTGTTAACCGACAGGAATTTTAACACTGCTTTTTT>Seq8_H.columella_ind1_WM [organism=Hemimycale columella] Individual1 Western Mediterranean SeaCTTCGCCAGGATCAATGCTAGGGGACGATCATTTGTATAACGTTCTTGTAACTGCGCATGCTTTTGTAATGATATTTTTTTTAGTTATGCCAGTAATGATTGGAGGGTTTGGTAACTGACTGGTGCCCTTGTATATTGGAGCGCCTGATATGGCTTTTCCTCGATTAAATAATATAAGTTTTTGATTATTGCCCCCGGCTTTAAGTCTATTGTTGGCGTCTGCTTTCGTGGAGCAAGGGGCAGGAACAGGATGAACTGTTTATCCTCCTCTCTCCGGGAATCAGGCTCATTCTGGCGGGTCAGTTGATTTAGTAATCTTTAGCCTACATTTAGCAGGAATTTCTTCAATTTTAGGCGCTATGAATTTTATTACTACAATAGTAAACATGCGGATAACGGGGATGACTTTTGATAGGATGCCTTTATTTGTTTGATCTATTTTAGTTACCGCGGTTTTATTGTTATTGTCCCTACCTGTATTAGCGGGGGCTATTACCATGTTGTTAACCGACAGGAATTTTAACACTGCTTTTTT>Seq9_H.columella_ind2_WM [organism=Hemimycale columella] Individual2 Western Mediterranean SeaCTTCGCCAGGATCAATGCTAGGGGACGATCATTTGTATAACGTTCTTGTAACTGCGCATGCTTTTGTAATGATATTTTTTTTAGTTATGCCAGTAATGATTGGAGGGTTTGGTAACTGACTGGTGCCCTTGTATATTGGAGCGCCTGATATGGCTTTTCCTCGATTAAATAATATAAGTTTTTGATTATTGCCCCCGGCTTTAAGTCTATTGTTGGCGTCTGCTTTCGTGGAGCAAGGGGCAGGAACAGGATGAACTGTTTATCCTCCTCTCTCCGGGAATCAGGCTCATTCTGGCGGGTCAGTTGATTTAGTAATCTTTAGCCTACATTTAGCAGGAATTTCTTCAATTTTAGGCGCTATGAATTTTATTACTACAATAGTAAACATGCGGATAACGGGGATGACTTTTGATAGGATGCCTTTATTTGTTTGATCTATTTTAGTTACCGCGGTTTTATTGTTATTGTCCCTACCTGTATTAGCGGGGGCTATTACCATGTTGTTAACCGACAGGAATTTTAACACTGCTTTTTT>Seq10_H.columella_ind3_WM [organism=Hemimycale columella] Individual3 Western Mediterranean SeaCTTCGCCAGGATCAATGCTAGGGGACGATCATTTGTATAACGTTCTTGTAACTGCGCATGCTTTTGTAATGATATTTTTTTTAGTTATGCCAGTAATGATTGGAGGGTTTGGTAACTGACTGGTGCCCTTGTATATTGGAGCGCCTGATATGGCTTTTCCTCGATTAAATAATATAAGTTTTTGATTATTGCCCCCGGCTTTAAGTCTATTGTTGGCGTCTGCTTTCGTGGAGCAAGGGGCAGGAACAGGATGAACTGTTTATCCTCCTCTCTCCGGGAATCAGGCTCATTCTGGCGGGTCAGTTGATTTAGTAATCTTTAGCCTACATTTAGCAGGAATTTCTTCAATTTTAGGCGCTATGAATTTTATTACTACAATAGTAAACATGCGGATAACGGGGATGACTTTTGATAGGATGCCTTTATTTGTTTGATCTATTTTAGTTACCGCGGTTTTATTGTTATTGTCCCTACCTGTATTAGCGGGGGCTATTACCATGTTGTTAACCGACAGGAATTTTAACACTGCTTTTTT>Seq11_H.mediterranea_ind1_WM [organism=Hemimycale mediterranea] Individual1 Western Mediterranean SeaCTTCGCCAGGATCAATGCTAGGGGACGATCATTTGTATAACGTTCTTGTAACTGCGCATGCTTTTGTAATGATATTTTTTTTAGTTATGCCAGTAATGATTGGAGGGTTTGGTAACTGACTGGTGCCTTTGTATATTGGAGCGCCTGATATGGCTTTTTCTCGATTAAATAATATAAGTTTTTGATTATTGCCCCCGGCTTTAAGTTTATTRTTGGCGTCTGCTTTCGTGGAGCAAGGGGCTGGAACAGGATGAACTGTTTATCCTCCTCTCTCTGGGAATCAGGCTCATTCTGGCGGGTCAGTTGATTTAGTAATCTTTAGCCTACATTTAGCAGGAATTTCTTCAATTTTAGGCGCTATGAATTTTATTACTACAATAGTAAACATGCGGATAACGGGGATGACTTTTGATAGGTTGCCTTTATTTGTTTGATCTATTTTAGTTACCGCAGTTTTATTGTTATTGTCCTTGCCTGTATTAGCGGGGGCTATTACCATGTTGTTAACCGATAGGAATTTTAACACTGCTTTTTT>Seq12_H.mediterranea_ind4_WM [organism=Hemimycale mediterranea] Individual4 Western Mediterranean SeaCTTCGCCAGGATCAATGCTAGGGGACGATCATTTGTATAACGTTCTTGTAACTGCGCATGCTTTTGTAATGATATTTTTTTTAGTTATGCCAGTAATGATTGGAGGGTTTGGTAACTGACTGGTGCCTTTGTATATTGGAGCGCCTGATATGGCTTTTCCTCGATTAAATAATATAAGTTTTTGATTATTGCCCCCGGCTTTAAGTTTATTGTTGGCGTCTGCTTTCGTGGAGCAAGGGGCTGGAACAGGATGAACTGTTTATCCTCCTCTCTCTGGGAATCAGGCTCATTCTGGCGGGTCAGTTGATTTAGTAATCTTTAGCCTACATTTAGCAGGAATTTCTTCAATTTTAGGCGCTATGAATTTTATTACTACAATAGTAAACATGCGGATAACGGGGATGACTTTTGATAGGTTGCCTTTATTTGTTTGATCTATTTTAGTTACCGCAGTTTTATTGTTATTGTCCTTGCCTGTATTAGCGGGGGCTATTACCATGTTGTTAACCGATAGGAATTTTAACACTGCTTTTTT>Seq13_H.mediterranea_ind5_WM [organism=Hemimycale mediterranea] Individual5 Western Mediterranean SeaCTTCGCCAGGATCAATGCTAGGGGACGATCATTTGTATAACGTTCTTGTAACTGCGCATGCTTTTGTAATGATATTTTTTTTAGTTATGCCAGTAATGATTGGAGGGTTTGGTAACTGACTGGTGCCTTTGTATATTGGAGCGCCTGATATGGCTTTTCCTCGATTAAATAATATAAGTTTTTGATTATTGCCCCCGGCTTTAAGTTTATTGTTGGCGTCTGCTTTCGTGGAGCAAGGGGCTGGAACAGGATGAACTGTTTATCCTCCTCTCTCTGGGAATCAGGCTCATTCTGGCGGGTCAGTTGATTTAGTAATCTTTAGCCTACATTTAGCAGGAATTTCTTCAATTTTAGGCGCTATGAATTTTATTACTACAATAGTAAACATGCGGATAACGGGGATGACTTTTGATAGGTTGCCTTTATTTGTTTGATCTATTTTAGTTACCGCAGTTTTATTGTTATTGTCCTTGCCTGTATTAGCGGGGGCTATTACCATGTTGTTAACCGATAGGAATTTTAACACTGCTTTTTT>Seq14_H.mediterranea_ind2_AS [organism=Hemimycale mediterranea] Individual2 Adriatic SeaCTTCGCCAGGATCAATGCTAGGGGACGATCATTTGTATAACGTTCTTGTAACTGCGCATGCTTTTGTAATGATATTTTTTTTAGTTATGCCAGTAATGATTGGAGGGTTTGGTAACTGACTGGTGCCTTTGTATATTGGAGCGCCTGATATGGCTTTTCCTCGATTAAATAATATAAGTTTTTGATTATTGCCCCCGGCTTTAAGTTTATTGTTGGCGTCTGCTTTCGTGGAGCAAGGGGCTGGAACAGGATGAACTGTTTATCCTCCTCTCTCTGGGAATCAGGCTCATTCTGGCGGGTCAGTTGATTTAGTAATCTTTAGCCTACATTTAGCAGGAATTTCTTCAATTTTAGGCGCTATGAATTTTATTACTACAATAGTAAACATGCGGATAACGGGGATGACTTTTGATAGGTTGCCTTTATTTGTTTGATCTATTTTAGTTACCGCAGTTTTATTGTTATTGTCCTTGCCTGTATTAGCGGGGGCTATTACCATGTTGTTAACCGATAGGAATTTTAACACTGCTTTTTT>Seq15_H.mediterranea_ind3_AS [organism=Hemimycale mediterranea] Individual3 Adriatic SeaCTTCGCCAGGATCAATGCTAGGGGACGATCATTTGTATAACGTTCTTGTAACTGCGCATGCTTTTGTAATGATATTTTTTTTAGTTATGCCAGTAATGATTGGAGGGTTTGGTAACTGACTGGTGCCTTTGTATATTGGAGCGCCTGATATGGCTTTTCCTCGATTAAATAATATAAGTTTTTGATTATTGCCCCCGGCTTTAAGTTTATTGTTGGCGTCTGCTTTCGTGGAGCAAGGGGCTGGAACAGGATGAACTGTTTATCCTCCTCTCTCTGGGAATCAGGCTCATTCTGGCGGGTCAGTTGATTTAGTAATCTTTAGCCTACATTTAGCAGGAATTTCTTCAATTTTAGGCGCTATGAATTTTATTACTACAATAGTAAACATGCGGATAACGGGGATGACTTTTGATAGGTTGCCTTTATTTGTTTGATCTATTTTAGTTACCGCAGTTTTATTGTTATTGTCCTTGCCTGTATTAGCGGGGGCTATTACCATGTTGTTAACCGATAGGAATTTTAACACTGCTTTTTT>Seq1 [organism=Crella cyathophora] Individual1 Red SeaTGGGTGGCAAACCCG-TGGGCGCAATGAAAGTGAAGGCAG-----GTGCTTGG-GGCCTGCTGT-GGCGAGAGCC-TCCG-----------GGCGCATCGTCGACCGATCCCGGG-CTACGCTGTGGCGGGATTTG-A-GTGAGAGCGTGCCTGTTGCGACCCGAAAGATGGTGAACTATGCCTGAATAGGGTGAAGCCAGAGGAAACCCTGGTGGAAGCTCGCAGCGATTCTGACGTGCAAATCGATCGTCAAATTTGGGTATAGGGGCGAAAGACTAATCGAACCGTCTAGTAGCTGGTTCCCTCCGAAGTTTCCCTCAGGATAGCTGGAGCCCGTGTTGGCAGTTTTATCAGGTAAAGCGAATGATTAGAGGTCTTGGGGCTGAAACGGCCTCAACCTATTCTCAAACTTTAAATGGGTAAGAAGCCCGGCTTGCTCGGTTGAAGTCGGGCGC-AGAATGCCTGGGCTCCCAGTGGGCCATTTTTGGTAAGCAGAACTGGCGATGTGGGATGAACCGAA-AGT--CGGGTTAAGGTGCCGGAATCGACGCTCATCAGATCCCATGAAAGGTGTTGGTTGATCCAGACAGCAGGACGGTGGCCATGGAAGTCGGAATCCGC>Seq2 [organism=Crella cyathophora] Individual2 Red SeaTGGGTGGCAAACCCG-TGGGCGCAATGAAAGTGAAGGCAG-----GTGCTTGG-GGCCTGCTGT-GGCGAGAGCC-TCCG-----------GGCGCATCGTCGACCGATCCCGGG-CTACGCTGTGGCGGGATTTG-A-GTGAGAGCGTGCCTGTTGCGACCCGAAAGATGGTGAACTATGCCTGAATAGGGTGAAGCCAGAGGAAACCCTGGTGGAAGCTCGCAGCGATTCTGACGTGCAAATCGATCGTCAAATTTGGGTATAGGGGCGAAAGACTAATCGAACCGTCTAGTAGCTGGTTCCCTCCGAAGTTTCCCTCAGGATAGCTGGAGCCCGTGTTGGCAGTTTTATCAGGTAAAGCGAATGATTAGAGGTCTTGGGGCTGAAACGGCCTCAACCTATTCTCAAACTTTAAATGGGTAAGAAGCCCGGCTTGCTCGGTTGAAGTCGGGCGC-AGAATGCCTGGGCTCCCAGTGGGCCATTTTTGGTAAGCAGAACTGGCGATGTGGGATGAACCGAA-AGT--CGGGTTAAGGTGCCGGAATCGACGCTCATCAGATCCCATGAAAGGTGTTGGTTGATCCAGACAGCAGGACGGTGGCCATGGAAGTCGGAATCCGC>Seq3 [organism=Crella cyathophora] Individual3 Indopacific OceanTGGGTGGCAAACCCG-TGGGCGCAATGAAAGTGAAGGCAG-----GCGCTCTG-GGCCTGCTGT-GGCGAGAGCC-GCTGCCCCCAGCTGCGGCGCATCGTCGACCGATCCCGGG-CTACGCTGTGGCGGGATTTG-A-GTGAGAGCGTGCCTGTTGCGACCCGAAAGATGGTGAACTATGCCTGAATAGGGTGAAGCCAGAGGAAACCCTGGTGGAAGCTCGCAGCGATTMTGACGTGCAAATCGATCGTCAAATTTGGGTATAGGGGCGAAAGACTAATCGAACCGTCTAGTAGCTGGTTCCCTCCGAAGTTTCCCTCAGGATAGCTGGAGCCCGT-GTGGCAGTTTTATCAGGTAAAGCGAATGATTAGAGGTCTTGGGGCTGAAACGGCCTCAACCTATTCTCAAACTTTAAATGGGTAAGAAGCCCGGCTTGCTCGGTTGAAGTCGGGCGC-AGAATGCCTGGGCTCCCAGTGGGCCATTTTTGGTAAGCAGAACTGGCGATGTGGGATGAACCGAA-AGT--CGGGTTAAGGTGCCGGAATCGACGCTCATCAGATCCCATGAAAGGTGTTGGTTGATACAGACAGCAGGACGGTGGCCATGGAAGTCGGAATCCGC>Seq4 [organism=Crella cyathophora] Individual4 Indopacific OceanTGGGTGGCAAACCCG-TGGGCGCAATGAAAGTGAAGGCAG-----GCGCTCTG-GGCCTGCTGT-GGCGAGAGCC-GCTGCCCCCAGCTGCGGCGCATCGTCGACCGATCCCGGG-CTACGCTGTGGCGGGATTTG-A-GTGAGAGCGTGCCTGTTGCGACCCGAAAGATGGTGAACTATGCCTGAATAGGGTGAAGCCAGAGGAAACCCTGGTGGAAGCTCGCAGCGATTCTGACGTGCAAATCGATCGTCAAATTTGGGTATAGGGGCGAAAGACTAATCGAACCGTCTAGTAGCTGGTTCCCTCCGAAGTTTCCCTCAGGATAGCTGGAGCCCGT-GTGGCAGTTTTATCAGGTAAAGCGAATGATTAGAGGTCTTGGGGCTGAAACGGCCTCAACCTATTCTCAAACTTTAAATGGGTAAGAAGCCCGGCTTGCTCGGTTGAAGTCGGGCGC-AGAATGCCTGGGCTCCCAGTGGGCCATTTTTGGTAAGCAGAACTGGCGATGTGGGATGAACCGAA-AGT--CGGGTTAAGGTGCCGGAATCGACGCTCATCAGATCCCATGAAAGGTGTTGGTTGATACAGACAGCAGGACGGTGGCCATGGAAGTCGGAATCCGC>Seq5 [organism=Hemimycale arabica] Individual1 Red SeaTGGGTGGCAAACCCG-TCGGCGCAATGAAAGTGAAGGCAG-----GTGTTCT--GGCCTGCTGT-GGCGAGAGCC-TCCCCGTCTCGTGGGGGCGCATCGTCGACCGATCCC-GAGCTACGCTGTGGCGGGATTTG-A-GTGAGAGCGTGCCTGTTGCGACCCGAAAGATGGTGAACTATGCCTGAATAGGGTGAAGCCAGAGGAAACCCTGGTGGAAGCTCGCAGCGATTCTGACGTGCAAATCGATCGTCAAATTTGGGTATAGGGGCGAAAGACTAATCGAACCGTCTAGTAGCTGGTTCCCTCCGAAGTTTCCCTCAGGATAGCTGGAGCCCG--TTGGCAGTTTTATCAGGTAAAGCGAATGATTAGAGGTCTTGGGGCTGAAACGGCCTCAACCTATTCTCAAACTTTAAATGGGTAAGAAGCCCGGCCTGCTCGGTTGAGGTCGGGCGC-AGAATGCCGGGGCTCCCAGTGGGCCATTTTTGGTAAGCAGAACTGGCGATGTGGGATGAACCGAA-AG--TCGGGTTAAGGTGCCGGAATCGACGCTCATCAGATCCCATGAAAGGTGTTGGTTGATACAGACAGCAGGACGGTGGCCATGGAAGTCGGAATCCGC>Seq6 [organism=Hemimycale arabica] Individual2 Red SeaTGGGTGGCAAACCCG-TCGGCGCAATGAAAGTGAAGGCAG-----GTGTTCT--GGCCTGCTGT-GGCGAGAGCC-TCCCCGTCTCGTGGGGGCGCATCGTCGACCGATCCC-GAGCTACGCTGTGGCGGGATTTG-A-GTGAGAGCGTGCCTGTTGCGACCCGAAAGATGGTGAACTATGCCTGAATAGGGTGAAGCCAGAGGAAACCCTGGTGGAAGCTCGCAGCGATTCTGACGTGCAAATCGATCGTCAAATTTGGGTATAGGGGCGAAAGACTAATCGAACCGTCTAGTAGCTGGTTCCCTCCGAAGTTTCCCTCAGGATAGCTGGAGCCCG--TTGGCAGTTTTATCAGGTAAAGCGAATGATTAGAGGTCTTGGGGCTGAAACGGCCTCAACCTATTCTCAAACTTTAAATGGGTAAGAAGCCCGGCCTGCTCGGTTGAGGTCGGGCGC-AGAATGCCGGGGCTCCCAGTGGGCCATTTTTGGTAAGCAGAACTGGCGATGTGGGATGAACCGAA-AG--TCGGGTTAAGGTGCCGGAATCGACGCTCATCAGATCCCATGAAAGGTGTTGGTTGATACAGACAGCAGGACGGTGGCCATGGAAGTCGGAATCCGC>Seq7[organism=Hemimycale columella] Individual1 Mediterranean Sea (Arenys)TGGGTGGCAAACCCG-TGGGCGCAATGAAAGTGAAGGCAG-----GTGTTCT--GGCCTGCCGT-GGCGGGAGCC-CTCGC-----GAGGGGGCGCACCGTCGACCGATCCC-AGGCTACGCTGTGGCGGGATTTG-A-GTGAGAGCGTGCCTGTTGCGACCCGAAAGATGGTGAACTATGCCTGAATAGGGTGAAGCCAGAGGAAACCCTGGTGGAAGCTCGCAGCGATTCTGACGTGCAAATCGATCGTCAAATTTGGGTATAGGGGCGAAAGACTAATCGAACCGTCTAGTAGCTGGTTCCCTCCGAAGTTTCCCTCAGGATAGCTGGAGCCCG--GTGGCAGTTTTATCAGGTAAAGCGAATGATTAGAGGTCTTGGGGCTGAAACGGCTTCAACCTATTCTCAAACTTTAAATGGGTAAGAAGCCCGGCCTGCTCGGTTGAGGTCGGGCACTAGAATGCCGGGGCTCCCAGTGGGCCATTTTTGGTAAGCAGAACTGGCGATGTGGGATGAACCGAA-AG--TCGGGTTAAGGTGCCGGAATCGACGCTCATCAGATCCCATGAAAGGTGTTGGTTGATACAGACAGCAGGACGGTGGCCATGGAAGTCGGAATCCGC>Seq8 [organism=Hemimycale columella] Individual2 Mediterranean Sea (Arenys)TGGGTGGCAAACCCG-TGGGCGCAATGAAAGTGAAGGCAG-----GTGTTCT--GGCCTGCCGT-GGCGGGAGCC-CTCGC-----GAGGGGGCGCACCGTCGACCGATCCC-AGGCTACGCTGTGGCGGGATTTG-A-GTGAGAGCGTGCCTGTTGCGACCCGAAAGATGGTGAACTATGCCTGAATAGGGTGAAGCCAGAGGAAACCCTGGTGGAAGCTCGCAGCGATTCTGACGTGCAAATCGATCGTCAAATTTGGGTATAGGGGCGAAAGACTAATCGAACCGTCTAGTAGCTGGTTCCCTCCGAAGTTTCCCTCAGGATAGCTGGAGCCCG--GTGGCAGTTTTATCAGGTAAAGCGAATGATTAGAGGTCTTGGGGCTGAAACGGCTTCAACCTATTCTCAAACTTTAAATGGGTAAGAAGCCCGGCCTGCTCGGTTGAGGTCGGGCACTAGAATGCCGGGGCTCCCAGTGGGCCATTTTTGGTAAGCAGAACTGGCGATGTGGGATGAACCGAA-AG--TCGGGTTAAGGTGCCGGAATCGACGCTCATCAGATCCCATGAAAGGTGTTGGTTGATACAGACAGCAGGACGGTGGCCATGGAAGTCGGAATCCGC>Seq9 [organism=Hemimycale columella] Individual1 Mediterranean SeaTGGGTGGCAAACCCG-TGGGCGCAATGAAAGTGAAGGCAG-----GCGTTCT--GGCCTGCTGT-GGCGGGAGCC-CTCGC-----GAGGGGGCGCACCGTCGACCGATCCC-AGGCTACGCTGTGGCGGGATTTG-A-GTGAGAGCGTGCCTGTTGCGACCCGAAAGATGGTGAACTATGCCTGAATAGGGTGAAGCCAGAGGAAACCCTGGTGGAAGCTCGCAGCGATTCTGACGTGCAAATCGATCGTCAAATTTGGGTATAGGGGCGAAAGACTAATCGAACCGTCTAGTAGCTGGTTCCCTCCGAAGTTTCCCTCAGGATAGCTGGAGCCCG--TAGGCAGTTTTATCAGGTAAAGCGAATGATTAGAGGTCTTGGGGCTGAAACGGCTTCAACCTATTCTCAAACTTTAAATGGGTAAGAAGCCCGGCCTGCTCGGTTGAGGTCGGGCACTAGAATGCCGGGGCTCCCAGTGGGCCATTTTTGGTAAGCAGAACTGGCGATGTGGGATGAACCGAA-AG--TCGGGTTAAGGTGCCGGAATCGACGCTCATCAGATCCCATGAAAGGTGTTGGTTGATACAGACAGCAGGACGGTGGCCATGGAAGTCGGAATCCGC>Seq10 [organism=Hemimycale columella] Individual2 Mediterranean SeaTGGGTGGCAAACCCG-TGGGCGCAATGAAAGTGAAGGCAG-----GCGTTCT--GGCCTGCTGT-GGCGGGAGCC-CTCGC-----GAGGGGGCGCACCGTCGACCGATCCC-AGGCTACGCTGTGGCGGGATTTG-A-GTGAGAGCGTGCCTGTTGCGACCCGAAAGATGGTGAACTATGCCTGAATAGGGTGAAGCCAGAGGAAACCCTGGTGGAAGCTCGCAGCGATTCTGACGTGCAAATCGATCGTCAAATTTGGGTATAGGGGCGAAAGACTAATCGAACCGTCTAGTAGCTGGTTCCCTCCGAAGTTTCCCTCAGGATAGCTGGAGCCCG--TAGGCAGTTTTATCAGGTAAAGCGAATGATTAGAGGTCTTGGGGCTGAAACGGCTTCAACCTATTCTCAAACTTTAAATGGGTAAGAAGCCCGGCCTGCTCGGTTGAGGTCGGGCACTAGAATGCCGGGGCTCCCAGTGGGCCATTTTTGGTAAGCAGAACTGGCGATGTGGGATGAACCGAA-AG--TCGGGTTAAGGTGCCGGAATCGACGCTCATCAGATCCCATGAAAGGTGTTGGTTGATACAGACAGCAGGACGGTGGCCATGGAAGTCGGAATCCGC>Seq11 [organism=Hemimycale columella] Individual3 Mediterranean SeaTGGGTGGCAAACCCG-TGGGCGCAATGAAAGTGAAGGCAG-----GCGTTCT--GGCCTGCTGT-GGCGGGAGCC-CTCGC-----GAGGGGGCGCACCGTCGACCGATCCC-AGGCTACGCTGTGGCGGGATTTG-A-GTGAGAGCGTGCCTGTTGCGACCCGAAAGATGGTGAACTATGCCTGAATAGGGTGAAGCCAGAGGAAACCCTGGTGGAAGCTCGCAGCGATTCTGACGTGCAAATCGATCGTCAAATTTGGGTATAGGGGCGAAAGACTAATCGAACCGTCTAGTAGCTGGTTCCCTCCGAAGTTTCCCTCAGGATAGCTGGAGCCCG--TWGGCAGTTTTATCAGGTAAAGCGAATGATTAGAGGTCTTGGGGCTGAAACGGCTTCAACCTATTCTCAAACTTTAAATGGGTAAGAAGCCCGGCCTGCTCGGTTGAGGTCGGGCACTAGAATGCCGGGGCTCCCAGTGGGCCATTTTTGGTAAGCAGAACTGGCGATGTGGGATGAACCGAA-AG--TCGGGTTAAGGTGCCGGAATCGACGCTCATCAGATCCCATGAAAGGTGTTGGTTGATACAGACAGCAGGACGGTGGCCATGGAAGTCGGAATCCGC>Seq12 [organism=Hemimycale columella] Individual4 Mediterranean SeaTGGGTGGCAAACCCG-TGGGCGCAATGAAAGTGAAGGCAG-----GCGTTCT--GGCCTGCTGT-GGCGGGAGCC-CTCGC-----GAGGGGGCGCACCGTCGACCGATCCC-AGGCTACGCTGTGGCGGGATTTG-A-GTGAGAGCGTGCCTGTTGCGACCCGAAAGATGGTGAACTATGCCTGAATAGGGTGAAGCCAGAGGAAACCCTGGTGGAAGCTCGCAGCGATTCTGACGTGCAAATCGATCGTCAAATTTGGGTATAGGGGCGAAAGACTAATCGAACCGTCTAGTAGCTGGTTCCCTCCGAAGTTTCCCTCAGGATAGCTGGAGCCCG--TAGGCAGTTTTATCAGGTAAAGCGAATGATTAGAGGTCTTGGGGCTGAAACGGCTTCAACCTATTCTCAAACTTTAAATGGGTAAGAAGCCCGGCCTGCTCGGTTGAGGTCGGGCACTAGAATGCCGGGGCTCCCAGTGGGCCATTTTTGGTAAGCAGAACTGGCGATGTGGGATGAACCGAA-AG--TCGGGTTAAGGTGCCGGAATCGACGCTCATCAGATCCCATGAAAGGTGTTGGTTGATACAGACAGCAGGACGGTGGCCATGGAAGTCGGAATCCGC>Seq13 [organism=Hemimycale mediterranea] Individual1 Mediterranean SeaTGGGTGGC-AACCCG-TCGGCGCAATGAAAGTGAAGGCAG-----GTGTTCC--GGCCTGCTGT-GGCGGGAGCC-CTCGC-----GAGGGGGCGCACCGTCGACCGATCCC-GGGCTACGCTGTGGCGGGATTTG-A-GTGAGAGCGTGCCTGTTGCGACCCGAAAGATGGTGAACTATGCCTGAATAGGGTGAAGCCAGAGGAAACCCTGGTGGAAGCTCGCAGCGATTCTGACGTGCAAATCGATCGTCAAATTTGGGTATAGGGGCGAAAGACTAATCGAACCGTCTAGTAGCTGGTTCCCTCCGAAGTTTCCCTCAGGATAGCTGGAGCCCG--TTGGCAGTTTTATCAGGTAAAGCGAATGATTAGAGGTCTTGGGGCTGAAACGGCTTCAACCTATTCTCAAACTTTAAATGGGTAAGAAGCCCGGCCTGCTCGGTTGAGGTCGGGCACTAGAATGCCGGGGCTCCCAGTGGGCCATTTTTGGTAAGCAGAACTGGCGATGTGGGATGAACCGAA-AG--TCGGGTTAAGGTGCCTGAATCGACGCTCATCAGATCCCATGAAAGGTGTTGGTTGATACAGACAGCAGGACGGTGGCCATGGAAGTCGGAACCCGC>Seq14 [organism=Hemimycale mediterranea] Individual2 Mediterranean SeaTGGGTGGCAAACCCG-TCGGCGCAATGAAAGTGAAGGCAG-----GTGTTCC--GGCCTGCTGT-GGCGGGAGCC-CTCGC-----GAGGGGGCGCACCGTCGACCGATCCC-GGGCTACGCTGTGGCGGGATTTG-A-GTGAGAGCGTGCCTGTTGCGACCCGAAAGATGGTGAACTATGCCTGAATAGGGTGAAGCCAGAGGAAACCCTGGTGGAAGCTCGCAGCGATTCTGACGTGCAAATCGATCGTCAAATTTGGGTATAGGGGCGAAAGACTAATCGAACCGTCTAGTAGCTGGTTCCCTCCGAAGTTTCCCTCAGGATAGCTGGAGCCCG--TTGGCAGTTTTATCAGGTAAAGCGAATGATTAGAGGTCTTGGGGCTGAAACGGCTTCAACCTATTCTCAAACTTTAAATGGGTAAGAAGCCCGGCCTGCTCGGTTGAGGTCGGGCACTAGAATGCCGGGGCTCCCAGTGGGCCATTTTTGGTAAGCAGAACTGGCGATGTGGGATGAACCGAA-AG--TCGGGTTAAGGTGCCTGAATCGACGCTCATCAGATCCCATGAAAGGTGTTGGTTGATACAGACAGCAGGACGGTGGCCATGGAAGTCGGAACCCGC>Seq15 [organism=Hemimycale mediterranea] Individual5 Adriatic SeaTGGGTGGCAAACCCG-TCGGCGCAATGAAAGTGAAGGCAG-----GTGTTCC--GGCCTGCTGT-GGCGGGAGCC-CTCGC-----GAGGGGGCGCACCGTCGACCGATCCC-GGGCTACGCTGTGGCGGGATTTG-A-GTGAGAGCGTGCCTGTTGCGACCCGAAAGATGGTGAACTATGCCTGAATAGGGTGAAGCCAGAGGAAACCCTGGTGGAAGCTCGCAGCGATTCTGACGTGCAAATCGATCGTCAAATTTGGGTATAGGGGCGAAAGACTAATCGAACCGTCTAGTAGCTGGTTCCCTCCGAAGTTTCCCTCAGGATAGCTGGAGCCCG--TTGGCAGTTTTATCAGGTAAAGCGAATGATTAGAGGTCTTGGGGCTGAAACGGCTTCAACCTATTCTCAAACTTTAAATGGGTAAGAAGCCCGGCCTGCTCGGTTGAGGTCGGGCACTAGAATGCCGGGGCTCCCAGTGGGCCATTTTTGGTAAGCAGAACTGGCGATGTGGGATGAACCGAA-AG--TCGGGTTAAGGTGCCTGAATCGACGCTCATCAGATCCCATGAAAGGTGTTGGTTGATACAGACAGCAGGACGGTGGCCATGGAAGTCGGAACCCGC>Seq16 [organism=Hemimycale mediterranea] Individual6 Adriatic SeaTGGGTGGCAAACCCG-TCGGCGCAATGAAAGTGAAGGCAG-----GTGTTCC--GGCCTGCTGT-GGCGGGAGCC-CTCGC-----GAGGGGGCGCACCGTCGACCGATCCC-GGGCTACGCTGTGGCGGGATTTG-A-GTGAGAGCGTGCCTGTTGCGACCCGAAAGATGGTGAACTATGCCTGAATAGGGTGAAGCCAGAGGAAACCCTGGTGGAAGCTCGCAGCGATTCTGACGTGCAAATCGATCGTCAAATTTGGGTATAGGGGCGAAAGACTAATCGAACCGTCTAGTAGCTGGTTCCCTCCGAAGTTTCCCTCAGGATAGCTGGAGCCCG--TTGGCAGTTTTATCAGGTAAAGCGAATGATTAGAGGTCTTGGGGCTGAAACGGCTTCAACCTATTCTCAAACTTTAAATGGGTAAGAAGCCCGGCCTGCTCGGTTGAGGTCGGGCACTAGAATGCCGGGGCTCCCAGTGGGCCATTTTTGGTAAGCAGAACTGGCGATGTGGGATGAACCGAA-AG--TCGGGTTAAGGTGCCTGAATCGACGCTCATCAGATCCCATGAAAGGTGTTGGTTGATACAGACAGCAGGACGGTGGCCATGGAAGTCGGAACCCGC>Seq17 [organism=Hemimycale mediterranea] Individual7 Adriatic SeaTGGGTGGCAAACCCG-TCGGCGCAATGAAAGTGAAGGCAG-----GTGTTCC--GGCCTGCTGT-GGCGGGAGCC-CTCGC-----GAGGGGGCGCACCGTCGACCGATCCC-GGGCTACGCTGTGGCGGGATTTG-A-GTGAGAGCGTGCCTGTTGCGACCCGAAAGATGGTGAACTATGCCTGAATAGGGTGAAGCCAGAGGAAACCCTGGTGGAAGCTCGCAGCGATTCTGACGTGCAAATCGATCGTCAAATTTGGGTATAGGGGCGAAAGACTAATCGAACCGTCTAGTAGCTGGTTCCCTCCGAAGTTTCCCTCAGGATAGCTGGAGCCCG--TTGGCAGTTTTATCAGGTAAAGCGAATGATTAGAGGTCTTGGGGCTGAAACGGCTTCAACCTATTCTCAAACTTTAAATGGGTAAGAAGCCCGGCCTGCTCGGTTGAGGTCGGGCACTAGAATGCCGGGGCTCCCAGTGGGCCATTTTTGGTAAGCAGAACTGGCGATGTGGGATGAACCGAA-AG--TCGGGTTAAGGTGCCTGAATCGACGCTCATCAGATCCCATGAAAGGTGTTGGTTGATACAGACAGCAGGACGGTGGCCATGGAAGTCGGAACCCGC>Seq18 [organism=Hemimycale mediterranea] Individual8 Adriatic SeaTGGGTGGCAAACCCG-TCGGCGCAATGAAAGTGAAGGCAG-----GTGTTCC--GGCCTGCTGT-GGCGGGAGCC-CTCGC-----GAGGGGGCGCACCGTCGACCGATCCC-GGGCTACGCTGTGGCGGGATTTG-A-GTGAGAGCGTGCCTGTTGCGACCCGAAAGATGGTGAACTATGCCTGAATAGGGTGAAGCCAGAGGAAACCCTGGTGGAAGCTCGCAGCGATTCTGACGTGCAAATCGATCGTCAAATTTGGGTATAGGGGCGAAAGACTAATCGAACCGTCTAGTAGCTGGTTCCCTCCGAAGTTTCCCTCAGGATAGCTGGAGCCCG--TTGGCAGTTTTATCAGGTAAAGCGAATGATTAGAGGTCTTGGGGCTGAAACGGCTTCAACCTATTCTCAAACTTTAAATGGGTAAGAAGCCCGGCCTGCTCGGTTGAGGTCGGGCACTAGAATGCCGGGGCTCCCAGTGGGCCATTTTTGGTAAGCAGAACTGGCGATGTGGGATGAACCGAA-AG--TCGGGTTAAGGTGCCTGAATCGACGCTCATCAGATCCCATGAAAGGTGTTGGTTGATACAGACAGCAGGACGGTGGCCATGGAAGTCGGAACCCGC>Seq19 [organism=Hemimycale mediterranea] Individual3 Mediterranean SeaTGGGTGGCAAACCCG-TCGGCGCAATGAAAGTGAAGGCAG-----GTGTTCC--GGCCTGCTGT-GGCGGGAGCC-CTCGC-----GAGGGGGCGCACCGTCGACCGATCCC-GGGCTACGCTGTGGCGGGATTTG-A-GTGAGAGCGTGCCTGTTGCGACCCGAAAGATGGTGAACTATGCCTGAATAGGGTGAAGCCAGAGGAAACCCTGGTGGAAGCTCGCAGCGATTCTGACGTGCAAATCGATCGTCAAATTTGGGTATAGGGGCGAAAGACTAATCGAACCGTCTAGTAGCTGGTTCCCTCCGAAGTTTCCCTCAGGATAGCTGGAGCCCG--TTGGCAGTTTTATCAGGTAAAGCGAATGATTAGAGGTCTTGGGGCTGAAACGGCTTCAACCTATTCTCAAACTTTAAATGGGTAAGAAGCCCGGCCTGCTCGGTTGAGGTCGGGCACTAGAATGCCGGGGCTCCCAGTGGGCCATTTTTGGTAAGCAGAACTGGCGATGTGGGATGAACCGAA-AG--TCGGGTTAAGGTGCCTGAATCGACGCTCATCAGATCCCATGAAAGGTGTTGGTTGATACAGACAGCAGGACGGTGGCCATGGAAGTCGGAACCCGC>Seq20 [organism=Hemimycale mediterranea] Individual4 Mediterranean SeaTGGGTGGCAAACCCG-TCGGCGCAATGAAAGTGAAGGCAG-----GTGTTCC--GGCCTGCTGT-GGCGGGAGCC-CTCGC-----GAGGGGGCGCACCGTCGACCGATCCC-GGGCTACGCTGTGGCGGGATTTG-A-GTGAGAGCGTGCCTGTTGCGACCCGAAAGATGGTGAACTATGCCTGAATAGGGTGAAGCCAGAGGAAACCCTGGTGGAAGCTCGCAGCGATTCTGACGTGCAAATCGATCGTCAAATTTGGGTATAGGGGCGAAAGACTAATCGAACCGTCTAGTAGCTGGTTCCCTCCGAAGTTTCCCTCAGGATAGCTGGAGCCCG--TTGGCAGTTTTATCAGGTAAAGCGAATGATTAGAGGTCTTGGGGCTGAAACGGCTTCAACCTATTCTCAAACTTTAAATGGGTAAGAAGCCCGGCCTGCTCGGTTGAGGTCGGGCACTAGAATGCCGGGGCTCCCAGTGGGCCATTTTTGGTAAGCAGAACTGGCGATGTGGGATGAACCGAA-AG--TCGGGTTAAGGTGCCTGAATCGACGCTCATCAGATCCCATGAAAGGTGTTGGTTGATACAGACAGCAGGACGGTGGCCATGGAAGTCGGAACCCGC>Seq21 [organism=Hemimycale mediterranea] Individual9 Adriatic SeaTGGGTGGCAAACCCG-TCGGCGCAATGAAAGTGAAGGCAG-----GTGTTCC--GGCCTGCTGT-GGCGGGAGCC-CTCGC-----GAGGGGGCGCACCGTCGACCGATCCC-GGGCTACGCTGTGGCGGGATTTG-A-GTGAGAGCGTGCCTGTTGCGACCCGAAAGATGGTGAACTATGCCTGAATAGGGTGAAGCCAGAGGAAACCCTGGTGGAAGCTCGCAGCGATTCTGACGTGCAAATCGATCGTCAAATTTGGGTATAGGGGCGAAAGACTAATCGAACCGTCTAGTAGCTGGTTCCCTCCGAAGTTTCCCTCAGGATAGCTGGAGCCCG--TTGGCAGTTTTATCAGGTAAAGCGAATGATTAGAGGTCTTGGGGCTGAAACGGCTTCAACCTATTCTCAAACTTTAAATGGGTAAGAAGCCCGGCCTGCTCGGTTGAGGTCGGGCACTAGAATGCCGGGGCTCCCAGTGGGCCATTTTTGGTAAGCAGAACTGGCGATGTGGGATGAACCGAA-AG--TCGGGTTAAGGTGCCTGAATCGACGCTCATCAGATCCCATGAAAGGTGTTGGTTGATACAGACAGCAGGACGGTGGCCATGGAAGTCGGAACCCGC>Seq22 [organism=Hemimycale mediterranea] Individual10 Adriatic SeaTGGGTGGCAAACCCG-TCGGCGCAATGAAAGTGAAGGCAG-----GTGTTCC--GGCCTGCTGT-GGCGGGAGCC-CTCGC-----GAGGGGGCGCACCGTCGACCGATCCC-GGGCTACGCTGTGGCGGGATTTG-A-GTGAGAGCGTGCCTGTTGCGACCCGAAAGATGGTGAACTATGCCTGAATAGGGTGAAGCCAGAGGAAACCCTGGTGGAAGCTCGCAGCGATTCTGACGTGCAAATCGATCGTCAAATTTGGGTATAGGGGCGAAAGACTAATCGAACCGTCTAGTAGCTGGTTCCCTCCGAAGTTTCCCTCAGGATAGCTGGAGCCCG--TTGGCAGTTTTATCAGGTAAAGCGAATGATTAGAGGTCTTGGGGCTGAAACGGCTTCAACCTATTCTCAAACTTTAAATGGGTAAGAAGCCCGGCCTGCTCGGTTGAGGTCGGGCACTAGAATGCCGGGGCTCCCAGTGGGCCATTTTTGGTAAGCAGAACTGGCGATGTGGGATGAACCGAA-AG--TCGGGTTAAGGTGCCTGAATCGACGCTCATCAGATCCCATGAAAGGTGTTGGTTGATACAGACAGCAGGACGGTGGCCATGGAAGTCGGAACCCGC>Seq23 [organism=Hemimycale mediterranea] Individual11 Adriatic SeaTGGGTGGCAAACCCG-TCGGCGCAATGAAAGTGAAGGCAG-----GTGTTCC--GGCCTGCTGT-GGCGGGAGCC-CTCGC-----GAGGGGGCGCACCGTCGACCGATCCC-GGGCTACGCTGTGGCGGGATTTG-A-GTGAGAGCGTGCCTGTTGCGACCCGAAAGATGGTGAACTATGCCTGAATAGGGTGAAGCCAGAGGAAACCCTGGTGGAAGCTCGCAGCGATTCTGACGTGCAAATCGATCGTCAAATTTGGGTATAGGGGCGAAAGACTAATCGAACCGTCTAGTAGCTGGTTCCCTCCGAAGTTTCCCTCAGGATAGCTGGAGCCCG--TTGGCAGTTTTATCAGGTAAAGCGAATGATTAGAGGTCTTGGGGCTGAAACGGCTTCAACCTATTCTCAAACTTTAAATGGGTAAGAAGCCCGGCCTGCTCGGTTGAGGTCGGGCACTAGAATGCCGGGGCTCCCAGTGGGCCATTTTTGGTAAGCAGAACTGGCGATGTGGGATGAACCGAA-AG--TCGGGTTAAGGTGCCTGAATCGACGCTCATCAGATCCCATGAAAGGTGTTGGTTGATACAGACAGCAGGACGGTGGCCATGGAAGTCGGAACCCGC>Seq1 [organism=Hemimycale columella] Individual1 Western Mediterranean SeaGGGAAGGGTGTATTTATTAGATCCAAAACCAGCGCGGGTCCTCCGGGTCCCGGTTGCCTGGCGACTCATGATAACTGCTCGAACCGTACGGCCGCCCGTGCCGACGGTGCTTCATTCAAATTTCTGCCCTATCAACTTTCGATGGTACGGTAGTGGCCTACCATGGTTGCAACGGGTGACGGAGAATTAGGGTTCGATTCCGGAGAGGGAGCCTGAGAGACGGCTACCACATCCAAGGAAGGCAGCAGGCGCGCAAATTACCCAATCCCGACTCGGGGAGGTAGTGACAATAAATAACAATGCCGGGCTATCGTAGTCTGGCAATTGGAATGAGTACAATCTAAACCCCTTAACGAGGAACAATTGGAGGGCAAGTCTGGTGCCAGCAGCCGCGGTAATTCCAGCTCCAATAGCGTATATTAAAGTTGTTGCAGTTAAAAAGCTCGTAGTTGGATTTCGGGGCGGCCTGGCCGGTCCGTCGCGAGACGAGTACTGGTCAGCCGCCCTTCCTCTCGAAAGCCCCGACTGCTCTTCGCTGCAGTGGTCGGGTAGTTCGGGACGTTTACTTTGAAAAAATTAGAGTGTTCAAGGCAGGCTGTCGCCTGGATACATTAGCATGGAATAATGGAAGAGGACCTCGGTCCTATTTTGTTGGTTTCTGGGGCCGAAGTAATGATTAAGAGGGACAGTTGGGG>Seq2 [organism=Hemimycale columella] Individual2 Western Mediterranean SeaGGGAAGGGTGTATTTATTAGATCCAAAACCAGCGCGGGTCCTCCGGGTCCCGGTTGCCTGGCGACTCATGATAACTGCTCGAACCGTACGGCCGCCCGTGCCGACGGTGCTTCATTCAAATTTCTGCCCTATCAACTTTCGATGGTACGGTAGTGGCCTACCATGGTTGCAACGGGTGACGGAGAATTAGGGTTCGATTCCGGAGAGGGAGCCTGAGAGACGGCTACCACATCCAAGGAAGGCAGCAGGCGCGCAAATTACCCAATCCCGACTCGGGGAGGTAGTGACAATAAATAACAATGCCGGGCTATCGTAGTCTGGCAATTGGAATGAGTACAATCTAAACCCCTTAACGAGGAACAATTGGAGGGCAAGTCTGGTGCCAGCAGCCGCGGTAATTCCAGCTCCAATAGCGTATATTAAAGTTGTTGCAGTTAAAAAGCTCGTAGTTGGATTTCGGGGCGGCCTGGCCGGTCCGTCGCGAGACGAGTACTGGTCAGCCGCCCTTCCTCTCGAAAGCCCCGACTGCTCTTCGCTGCAGTGGTCGGGTAGTTCGGGACGTTTACTTTGAAAAAATTAGAGTGTTCAAGGCAGGCTGTCGCCTGGATACATTAGCATGGAATAATGGAAGAGGACCTCGGTCCTATTTTGTTGGTTTCTGGGGCCGAAGTAATGATTAAGAGGGACAGTTGGGG>Seq3 [organism=Hemimycale mediterranea] Individual1 Western Mediterranean SeaGGGAAGGGTGTATTTATTAGATCCAAAACCAGCGCGGGTCCTCCGGGTCCCGGTTGCCTGGCGACTCATGATAACTGCTCGAACCGCATGGCCTCCAGTGCCGGCGGTGCTTCATTCAAATTTCTGCCCTATCAACTTTCGATGGTACGGTAGTGGCCTACCATGGTTGCAACGGGTGACGGAGAATTAGGGTTCGATTCCGGAGAGGGAGCCTGAGAGACGGCTACCACATCCAAGGAAGGCAGCAGGCGCGCAAATTACCCAATCCCGACTCGGGGAGGTAGTGACAATAAATAACAATGCCGGACTATCGTAGTCTGGCAATTGGAATGAGTACAATCTAAACCCCTTAACGAGGAACAATTGGAGGGCAAGTCTGGTGCCAGCAGCCGCGGTAATTCCAGCTCCAATAGCGTATATTAAAGTTGTTGCAGTTAAAAAGCTCGTAGTTGGATTTCGGGGCGGCCTGGCCGGTCCGTCGCGAGACGAGTACTGGTCAGCCGCCCTTCCTCTCGAAAGCCCCGACTGCTCTTCGCTGCAGTGGTCGGGTAGTTCGGGACGTTTACTTTGAAAAAATTAGAGTGTTCAAGGCAGGCTGTCGCCTGGATACATTAGCATGGAATAATGGAAGAGGACCTCGGTCCTATTTTGTTGGTTTCTGGGGCCGAAGTAATGATTAAGAGGGACAGTTGGGG>Seq4 [organism=Hemimycale mediterranea] Individual2 Western Mediterranean SeaGGGAAGGGTGTATTTATTAGATCCAAAACCAGCGCGGGTCCTCCGGGTCCCGGTTGCCTGGCGACTCATGATAACTGCTCGAACCGCATGGCCTCCAGTGCCGGCGGTGCTTCATTCAAATTTCTGCCCTATCAACTTTCGATGGTACGGTAGTGGCCTACCATGGTTGCAACGGGTGACGGAGAATTAGGGTTCGATTCCGGAGAGGGAGCCTGAGAGACGGCTACCACATCCAAGGAAGGCAGCAGGCGCGCAAATTACCCAATCCCGACTCGGGGAGGTAGTGACAATAAATAACAATGCCGGACTATCGTAGTCTGGCAATTGGAATGAGTACAATCTAAACCCCTTAACGAGGAACAATTGGAGGGCAAGTCTGGTGCCAGCAGCCGCGGTAATTCCAGCTCCAATAGCGTATATTAAAGTTGTTGCAGTTAAAAAGCTCGTAGTTGGATTTCGGGGCGGCCTGGCCGGTCCGTCGCGAGACGAGTACTGGTCAGCCGCCCTTCCTCTCGAAAGCCCCGACTGCTCTTCGCTGCAGTGGTCGGGTAGTTCGGGACGTTTACTTTGAAAAAATTAGAGTGTTCAAGGCAGGCTGTCGCCTGGATACATTAGCATGGAATAATGGAAGAGGACCTCGGTCCTATTTTGTTGGTTTCTGGGGCCGAAGTAATGATTAAGAGGGACAGTTGGGG>Seq5 [organism=Hemimycale mediterranea] Individual8 Adriatic SeaGGGAAGGGTGTATTTATTAGATCCAAAACCAGCGCGGGTCCTCCGGGTCCCGGTTGCCTGGCGACTCATGATAACTGCTCGAACCGCATGGCCTCCAGTGCCGGCGGTGCTTCATTCAAATTTCTGCCCTATCAACTTTCGATGGTACGGTAGTGGCCTACCATGGTTGCAACGGGTGACGGAGAATTAGGGTTCGATTCCGGAGAGGGAGCCTGAGAGACGGCTACCACATCCAAGGAAGGCAGCAGGCGCGCAAATTACCCAATCCCGACTCGGGGAGGTAGTGACAATAAATAACAATGCCGGACTATCGTAGTCTGGCAATTGGAATGAGTACAATCTAAACCCCTTAACGAGGAACAATTGGAGGGCAAGTCTGGTGCCAGCAGCCGCGGTAATTCCAGCTCCAATAGCGTATATTAAAGTTGTTGCAGTTAAAAAGCTCGTAGTTGGATTTCGGGGCGGCCTGGCCGGTCCGTCGCGAGACGAGTACTGGTCAGCCGCCCTTCCTCTCGAAAGCCCCGACTGCTCTTCGCTGCAGTGGTCGGGTAGTTCGGGACGTTTACTTTGAAAAAATTAGAGTGTTCAAGGCAGGCTGTCGCCTGGATACATTAGCATGGAATAATGGAAGAGGACCTCGGTCCTATTTTGTTGGTTTCTGGGGCCGAAGTAATGATTAAGAGGGACAGTTGGGG>Seq6 [organism=Hemimycale mediterranea] Individual9 Adriatic SeaGGGAAGGGTGTATTTATTAGATCCAAAACCAGCGCGGGTCCTCCGGGTCCCGGTTGCCTGGCGACTCATGATAACTGCTCGAACCGCATGGCCTCCAGTGCCGGCGGTGCTTCATTCAAATTTCTGCCCTATCAACTTTCGATGGTACGGTAGTGGCCTACCATGGTTGCAACGGGTGACGGAGAATTAGGGTTCGATTCCGGAGAGGGAGCCTGAGAGACGGCTACCACATCCAAGGAAGGCAGCAGGCGCGCAAATTACCCAATCCCGACTCGGGGAGGTAGTGACAATAAATAACAATGCCGGACTATCGTAGTCTGGCAATTGGAATGAGTACAATCTAAACCCCTTAACGAGGAACAATTGGAGGGCAAGTCTGGTGCCAGCAGCCGCGGTAATTCCAGCTCCAATAGCGTATATTAAAGTTGTTGCAGTTAAAAAGCTCGTAGTTGGATTTCGGGGCGGCCTGGCCGGTCCGTCGCGAGACGAGTACTGGTCAGCCGCCCTTCCTCTCGAAAGCCCCGACTGCTCTTCGCTGCAGTGGTCGGGTAGTTCGGGACGTTTACTTTGAAAAAATTAGAGTGTTCAAGGCAGGCTGTCGCCTGGATACATTAGCATGGAATAATGGAAGAGGACCTCGGTCCTATTTTGTTGGTTTCTGGGGCCGAAGTAATGATTAAGAGGGACAGTTGGGG>Seq7 [organism=Hemimycale mediterranea] Individual5 Adriatic SeaGGGAAGGGTGTATTTATTAGATCCAAAACCAGCGCGGGTCCTCCGGGTCCCGGTTGCCTGGCGACTCATGATAACTGCTCGAACCGCATGGCCTCCAGTGCCGGCGGTGCTTCATTCAAATTTCTGCCCTATCAACTTTCGATGGTACGGTAGTGGCCTACCATGGTTGCAACGGGTGACGGAGAATTAGGGTTCGATTCCGGAGAGGGAGCCTGAGAGACGGCTACCACATCCAAGGAAGGCAGCAGGCGCGCAAATTACCCAATCCCGACTCGGGGAGGTAGTGACAATAAATAACAATGCCGGACTATCGTAGTCTGGCAATTGGAATGAGTACAATCTAAACCCCTTAACGAGGAACAATTGGAGGGCAAGTCTGGTGCCAGCAGCCGCGGTAATTCCAGCTCCAATAGCGTATATTAAAGTTGTTGCAGTTAAAAAGCTCGTAGTTGGATTTCGGGGCGGCCTGGCCGGTCCGTCGCGAGACGAGTACTGGTCAGCCGCCCTTCCTCTCGAAAGCCCCGACTGCTCTTCGCTGCAGTGGTCGGGTAGTTCGGGACGTTTACTTTGAAAAAATTAGAGTGTTCAAGGCAGGCTGTCGCCTGGATACATTAGCATGGAATAATGGAAGAGGACCTCGGTCCTATTTTGTTGGTTTCTGGGGCCGAAGTAATGATTAAGAGGGACAGTTGGGG>Seq8 [organism=Hemimycale mediterranea] Individual6 Adriatic SeaGGGAAGGGTGTATTTATTAGATCCAAAACCAGCGCGGGTCCTCCGGGTCCCGGTTGCCTGGCGACTCATGATAACTGCTCGAACCGCATGGCCTCCAGTGCCGGCGGTGCTTCATTCAAATTTCTGCCCTATCAACTTTCGATGGTACGGTAGTGGCCTACCATGGTTGCAACGGGTGACGGAGAATTAGGGTTCGATTCCGGAGAGGGAGCCTGAGAGACGGCTACCACATCCAAGGAAGGCAGCAGGCGCGCAAATTACCCAATCCCGACTCGGGGAGGTAGTGACAATAAATAACAATGCCGGACTATCGTAGTCTGGCAATTGGAATGAGTACAATCTAAACCCCTTAACGAGGAACAATTGGAGGGCAAGTCTGGTGCCAGCAGCCGCGGTAATTCCAGCTCCAATAGCGTATATTAAAGTTGTTGCAGTTAAAAAGCTCGTAGTTGGATTTCGGGGCGGCCTGGCCGGTCCGTCGCGAGACGAGTACTGGTCAGCCGCCCTTCCTCTCGAAAGCCCCGACTGCTCTTCGCTGCAGTGGTCGGGTAGTTCGGGACGTTTACTTTGAAAAAATTAGAGTGTTCAAGGCAGGCTGTCGCCTGGATACATTAGCATGGAATAATGGAAGAGGACCTCGGTCCTATTTTGTTGGTTTCTGGGGCCGAAGTAATGATTAAGAGGGACAGTTGGGG>Seq9 [organism=Hemimycale mediterranea] Individual3 Mediterranean SeaGGGAAGGGTGTATTTATTAGATCCAAAACCAGCGCGGGTCCTCCGGGTCCCGGTTGCCTGGCGACTCATGATAACTGCTCGAACCGCATGGCCTCCAGTGCCGGCGGTGCTTCATTCAAATTTCTGCCCTATCAACTTTCGATGGTACGGTAGTGGCCTACCATGGTTGCAACGGGTGACGGAGAATTAGGGTTCGATTCCGGAGAGGGAGCCTGAGAGACGGCTACCACATCCAAGGAAGGCAGCAGGCGCGCAAATTACCCAATCCCGACTCGGGGAGGTAGTGACAATAAATAACAATGCCGGACTATCGTAGTCTGGCAATTGGAATGAGTACAATCTAAACCCCTTAACGAGGAACAATTGGAGGGCAAGTCTGGTGCCAGCAGCCGCGGTAATTCCAGCTCCAATAGCGTATATTAAAGTTGTTGCAGTTAAAAAGCTCGTAGTTGGATTTCGGGGCGGCCTGGCCGGTCCGTCGCGAGACGAGTACTGGTCAGCCGCCCTTCCTCTCGAAAGCCCCGACTGCTCTTCGCTGCAGTGGTCGGGTAGTTCGGGACGTTTACTTTGAAAAAATTAGAGTGTTCAAGGCAGGCTGTCGCCTGGATACATTAGCATGGAATAATGGAAGAGGACCTCGGTCCTATTTTGTTGGTTTCTGGGGCCGAAGTAATGATTAAGAGGGACAGTTGGGG>Seq10 [organism=Hemimycale mediterranea] Individual4 Mediterranean SeaGGGAAGGGTGTATTTATTAGATCCAAAACCAGCGCGGGTCCTCCGGGTCCCGGTTGCCTGGCGACTCATGATAACTGCTCGAACCGCATGGCCTCCAGTGCCGGCGGTGCTTCATTCAAATTTCTGCCCTATCAACTTTCGATGGTACGGTAGTGGCCTACCATGGTTGCAACGGGTGACGGAGAATTAGGGTTCGATTCCGGAGAGGGAGCCTGAGAGACGGCTACCACATCCAAGGAAGGCAGCAGGCGCGCAAATTACCCAATCCCGACTCGGGGAGGTAGTGACAATAAATAACAATGCCGGACTATCGTAGTCTGGCAATTGGAATGAGTACAATCTAAACCCCTTAACGAGGAACAATTGGAGGGCAAGTCTGGTGCCAGCAGCCGCGGTAATTCCAGCTCCAATAGCGTATATTAAAGTTGTTGCAGTTAAAAAGCTCGTAGTTGGATTTCGGGGCGGCCTGGCCGGTCCGTCGCGAGACGAGTACTGGTCAGCCGCCCTTCCTCTCGAAAGCCCCGACTGCTCTTCGCTGCAGTGGTCGGGTAGTTCGGGACGTTTACTTTGAAAAAATTAGAGTGTTCAAGGCAGGCTGTCGCCTGGATACATTAGCATGGAATAATGGAAGAGGACCTCGGTCCTATTTTGTTGGTTTCTGGGGCCGAAGTAATGATTAAGAGGGACAGTTGGGG>Seq11 [organism=Hemimycale mediterranea] Individual7 Adriatic SeaGGGAAGGGTGTATTTATTAGATCCAAAACCAGCGCGGGTCCTCCGGGTCCCGGTTGCCTGGCGACTCATGATAACTGCTCGAACCGCATGGCCTCCAGTGCCGGCGGTGCTTCATTCAAATTTCTGCCCTATCAACTTTCGATGGTACGGTAGTGGCCTACCATGGTTGCAACGGGTGACGGAGAATTAGGGTTCGATTCCGGAGAGGGAGCCTGAGAGACGGCTACCACATCCAAGGAAGGCAGCAGGCGCGCAAATTACCCAATCCCGACTCGGGGAGGTAGTGACAATAAATAACAATGCCGGACTATCGTAGTCTGGCAATTGGAATGAGTACAATCTAAACCCCTTAACGAGGAACAATTGGAGGGCAAGTCTGGTGCCAGCAGCCGCGGTAATTCCAGCTCCAATAGCGTATATTAAAGTTGTTGCAGTTAAAAAGCTCGTAGTTGGATTTCGGGGCGGCCTGGCCGGTCCGTCGCGAGACGAGTACTGGTCAGCCGCCCTTCCTCTCGAAAGCCCCGACTGCTCTTCGCTGCAGTGGTCGGGTAGTTCGGGACGTTTACTTTGAAAAAATTAGAGTGTTCAAGGCAGGCTGTCGCCTGGATACATTAGCATGGAATAATGGAAGAGGACCTCGGTCCTATTTTGTTGGTTTCTGGGGCCGAAGTAATGATTAAGAGGGACAGTTGGGG>Seq12 [organism=Hemimycale arabica] Individual1 Red Sea GGAAGGGATGTATTTATTAGATCCAAAACCAGCGCGGGTCCTCCGGGTCCCGGTTGCCTGGCGATTCATGATAACTGCTCGAACCGTATGGCCCCC-GCGCCGACGGTGCTTCATTCAAATTTCTGCCCTATCAACTTTCGATGGTACGGTAGTGGCCTACCATGGTTGCAACGGGTGACGGAGAATTAGGGTTCGATTCCGGAGAGGGAGCCTGAGAGACGGCTACCACATCCAAGGAAGGCAGCAGGCGCGCAAATTACCCAATCCCGACTCGGGGAGGTAGTGACAATAAATAACAATGCCGGGCTATCGTAGTCTGGCAATTGGAATGAGTACAATCTAAACCCCTTAACGAGGAACAATTGGAGGGCAAGTCTGGTGCCAGCAGCCGCGGTAATTCCAGCTCCAATAGCGTATATTAAAGTTGTTGCAGTTAAAAAGCTCGTAGTTGGATTTCGGGGCGGCCTGGCTGGTCCGTCGCGAGACGAGTACTGGTCAGCCGCCCTTCCTCTCGAAAGCCCCGACTGCTCTTCACTGCAGTGGTCGGGTAGTTCGGGACGTTTACTTTGAAAAAATTAGAGTGTTCAAGGCAGGCTGTCGCCTGAATACATTAGCATGGAATAATGGAAGAGGACCTCGGTCCTATTTTGTTGGTTTCTGGGGCCGAAGTAATGATTAAGAGGGACAGTTGGGG>Seq13 [organism=Hemimycale arabica] Individual2 Red Sea GGAAGGGATGTATTTATTAGATCCAAAACCAGCGCGGGTCCTCCGGGTCCCGGTTGCCTGGCGATTCATGATAACTGCTCGAACCGTATGGCCCCC-GCGCCGACGGTGCTTCATTCAAATTTCTGCCCTATCAACTTTCGATGGTACGGTAGTGGCCTACCATGGTTGCAACGGGTGACGGAGAATTAGGGTTCGATTCCGGAGAGGGAGCCTGAGAGACGGCTACCACATCCAAGGAAGGCAGCAGGCGCGCAAATTACCCAATCCCGACTCGGGGAGGTAGTGACAATAAATAACAATGCCGGGCTATCGTAGTCTGGCAATTGGAATGAGTACAATCTAAACCCCTTAACGAGGAACAATTGGAGGGCAAGTCTGGTGCCAGCAGCCGCGGTAATTCCAGCTCCAATAGCGTATATTAAAGTTGTTGCAGTTAAAAAGCTCGTAGTTGGATTTCGGGGCGGCCTGGCTGGTCCGTCGCGAGACGAGTACTGGTCAGCCGCCCTTCCTCTCGAAAGCCCCGACTGCTCTTCACTGCAGTGGTCGGGTAGTTCGGGACGTTTACTTTGAAAAAATTAGAGTGTTCAAGGCAGGCTGTCGCCTGAATACATTAGCATGGAATAATGGAAGAGGACCTCGGTCCTATTTTGTTGGTTTCTGGGGCCGAAGTAATGATTAAGAGGGACAGTTGGGG>Seq14 [organism=Crella cyathophora] Individual1 Red Sea GGGAGGGATGTATTTATTAGATCCAAAACCAGCGCGGGTCCTCCGGGTCCCGGTTGCCTGGCGAGTCATGATAACTGCTCGAACCGTATGGCCCTC-GCGCCGACGGTGCTTCATTCAAATTTCTGCCCTATCAACTTTCGATGGTACGGTAGTGGCCTACCATGGTTGCAACGGGTGACGGAGAATTAGGGTTCGATTCCGGAGAGGGAGCCTGAGAGACGGCTACCACATCCAAGGAAGGCAGCAGGCGCGCAAATTACCCAATCCCGACTCGGGGAGGTAGTGACAATAAATAACAATGCCGGGCTATCGTAGTCTGGCAATTGGAATGAGTACAATCTAAACCCCTTAACGAGGAACAATTGGAGGGCAAGTCTGGTGCCAGCAGCCGCGGTAATTCCAGCTCCAATAGCGTATATTAAAGTTGTTGCAGTTAAAAAGCTCGTAGTTGGATTTCGGGGCGGCCTGGCTGGTCCGTCGCGAGACGAGCACTGGTCAGCCGCCCTTCCTCTCGAAAGCCCCGACTGCTCTTCACTGCAGTGGTCGGGTAGTTCGGGACGTTTACTTTGAAAAAATTAGAGTGTTCAAGGCAGGCTGTGGCCTGAATACATTAGCATGGAATAATGGAAGAGGACCTCGGTCCTATTTTGTTGGTTTCTGGGGCCGAAGTAATGATTAAGAGGGACAGTTGGGG>Seq15 [organism=Crella cyathophora] Individual2 Red Sea GGGAGGGATGTATTTATTAGATCCAAAACCAGCGCGGGTCCTCCGGGTCCCGGTTGCCTGGCGAGTCATGATAACTGCTCGAACCGTATGGCCCTC-GCGCCGACGGTGCTTCATTCAAATTTCTGCCCTATCAACTTTCGATGGTACGGTAGTGGCCTACCATGGTTGCAACGGGTGACGGAGAATTAGGGTTCGATTCCGGAGAGGGAGCCTGAGAGACGGCTACCACATCCAAGGAAGGCAGCAGGCGCGCAAATTACCCAATCCCGACTCGGGGAGGTAGTGACAATAAATAACAATGCCGGGCTATCGTAGTCTGGCAATTGGAATGAGTACAATCTAAACCCCTTAACGAGGAACAATTGGAGGGCAAGTCTGGTGCCAGCAGCCGCGGTAATTCCAGCTCCAATAGCGTATATTAAAGTTGTTGCAGTTAAAAAGCTCGTAGTTGGATTTCGGGGCGGCCTGGCTGGTCCGTCGCGAGACGAGCACTGGTCAGCCGCCCTTCCTCTCGAAAGCCCCGACTGCTCTTCACTGCAGTGGTCGGGTAGTTCGGGACGTTTACTTTGAAAAAATTAGAGTGTTCAAGGCAGGCTGTGGCCTGAATACATTAGCATGGAATAATGGAAGAGGACCTCGGTCCTATTTTGTTGGTTTCTGGGGCCGAAGTAATGATTAAGAGGGACAGTTGGGG>Seq16 [organism=Crella cyathophora] Individual3 Indopacfic OceanGGGAGGGATGTATTTATTAGATCCAAAACCAGCGCGGGTCCTCCGGGTCCCGGTTGCCTGGCGAGTCATGATAACTGCTCGAACCGTATGGCCCTC-GCGCCGACGGTGCTTCATTCAAATTTCTGCCCTATCAACTTTCGATGGTACGGTAGTGGCCTACCATGGTTGCAACGGGTGACGGAGAATTAGGGTTCGATTCCGGAGAGGGAGCCTGAGAGACGGCTACCACATCCAAGGAAGGCAGCAGGCGCGCAAATTACCCAATCCCGACTCGGGGAGGTAGTGACAATAAATAACAATGCCGGGCTATCGTAGTCTGGCAATTGGAATGAGTACAATCTAAACCCCTTAACGAGGAACAATTGGAGGGCAAGTCTGGTGCCAGCAGCCGCGGTAATTCCAGCTCCAATAGCGTATATTAAAGTTGTTGCAGTTAAAAAGCTCGTAGTTGGATTTCGGGGCGGCCTGGCTGGTCCGTCGCGAGACGAGCACTGGTCAGCCGCCCTTCCTCTCGAAAGCCCCGACTGCTCTTCACTGCAGTGGTCGGGTAGTTCGGGACGTTTACTTTGAAAAAATTAGAGTGTTCAAGGCAGGCTGTGGCCTGAATACATTAGCATGGAATAATGGAAGAGGACCTCGGTCCTATTTTGTTGGTTTCTGGGGCCGAAGTAATGATTAAGAGGGACAGTTGGGG>Seq17 [organism=Crella cyathophora] Individual4 Indopacific OceanGGGAGGGATGTATTTATTAGATCCAAAACCAGCGCGGGTCCTCCGGGTCCCGGTTGCCTGGCGAGTCATGATAACTGCTCGAACCGTATGGCCCTC-GCGCCGACGGTGCTTCATTCAAATTTCTGCCCTATCAACTTTCGATGGTACGGTAGTGGCCTACCATGGTTGCAACGGGTGACGGAGAATTAGGGTTCGATTCCGGAGAGGGAGCCTGAGAGACGGCTACCACATCCAAGGAAGGCAGCAGGCGCGCAAATTACCCAATCCCGACTCGGGGAGGTAGTGACAATAAATAACAATGCCGGGCTATCGTAGTCTGGCAATTGGAATGAGTACAATCTAAACCCCTTAACGAGGAACAATTGGAGGGCAAGTCTGGTGCCAGCAGCCGCGGTAATTCCAGCTCCAATAGCGTATATTAAAGTTGTTGCAGTTAAAAAGCTCGTAGTTGGATTTCGGGGCGGCCTGGCTGGTCCGTCGCGAGACGAGCACTGGTCAGCCGCCCTTCCTCTCGAAAGCCCCGACTGCTCTTCACTGCAGTGGTCGGGTAGTTCGGGACGTTTACTTTGAAAAAATTAGAGTGTTCAAGGCAGGCTGTGGCCTGAATACATTAGCATGGAATAATGGAAGAGGACCTCGGTCCTATTTTGTTGGTTTCTGGGGCCGAAGTAATGATTAAGAGGGACAGTTGGGG
