# Supplementary figures and images for: Molecular phylogenies confirm the presence of two cryptic Hemimycale species in the Mediterranean and reveal the polyphyly of the genera Crella and Hemimycale (Demospongiae: Poecilosclerida)
Source: PeerJ. 2017 Mar 7;5:e2958. doi: 10.7717/peerj.2958 (PMC5344016; doi:10.7717/peerj.2958)

18S

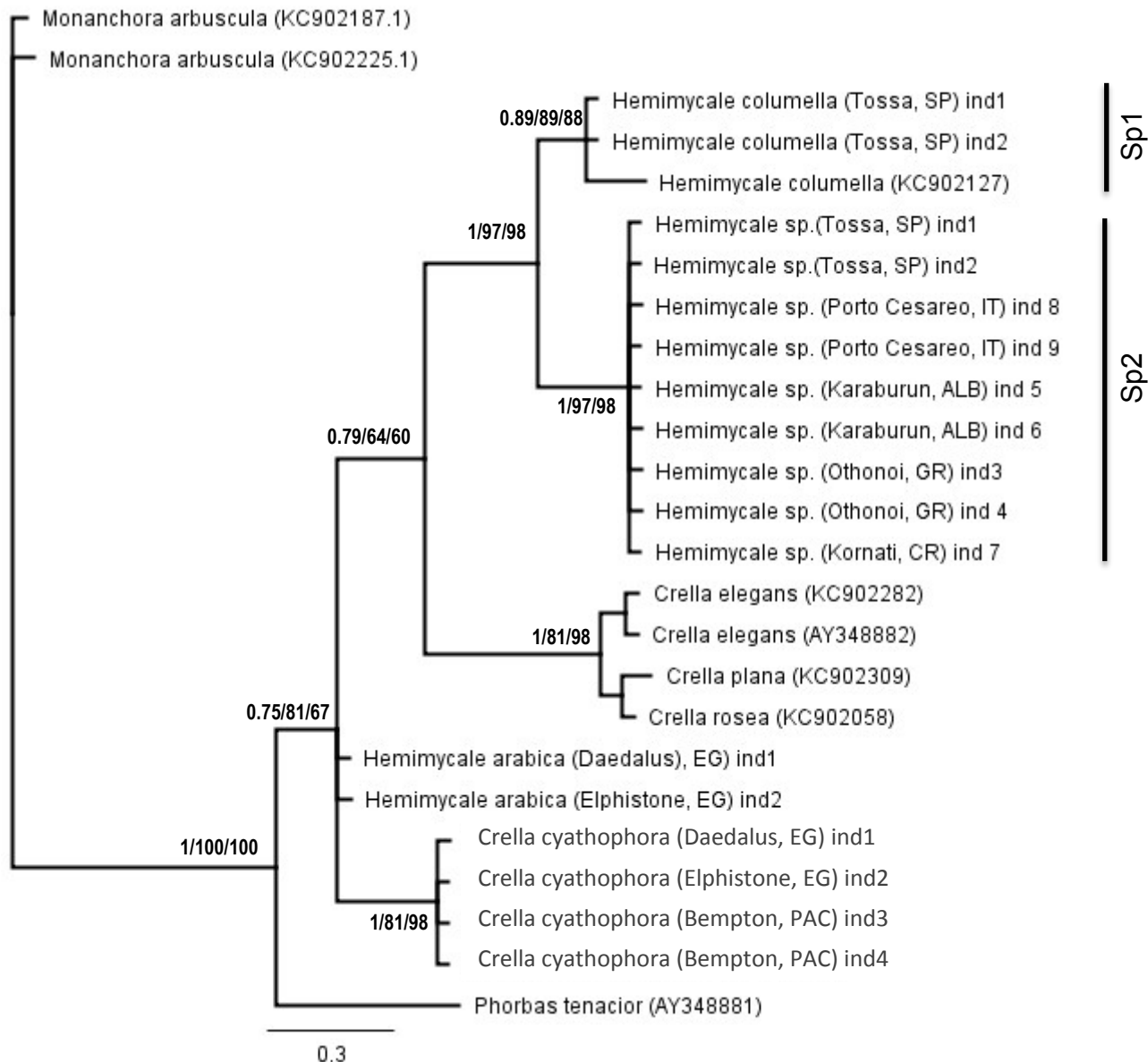

BI/NJ/ML

Supplement: Supplemental Information 2 — BI, NJ and ML gave almost the same topologies. The two individuals of H. arabica that appeared in unresolved positions under BI and ML formed a poorly supported (75%) clade in the tree under the NJ criterion (not shown). Posterior probability, neighbor joining and maximum likelihood supporting values are at the base of clades. [file peerj-05-2958-s002.pdf]

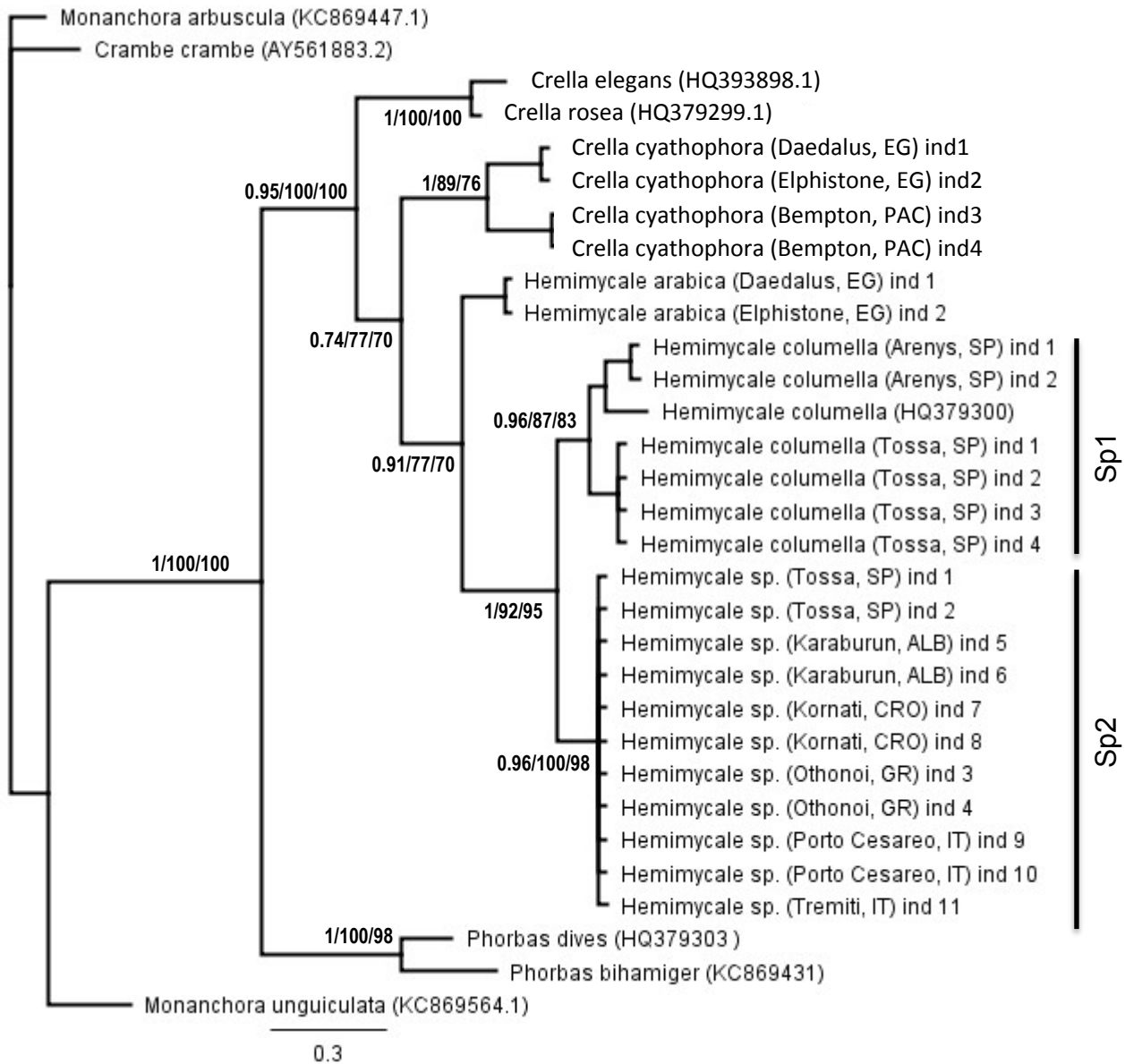

Supplement: Supplemental Information 3 — BI, NJ and ML gave congruent topologies. Posterior probability, neighbor joining and maximum likelihood supporting values are at the base of clades. [file peerj-05-2958-s003.pdf]

COI

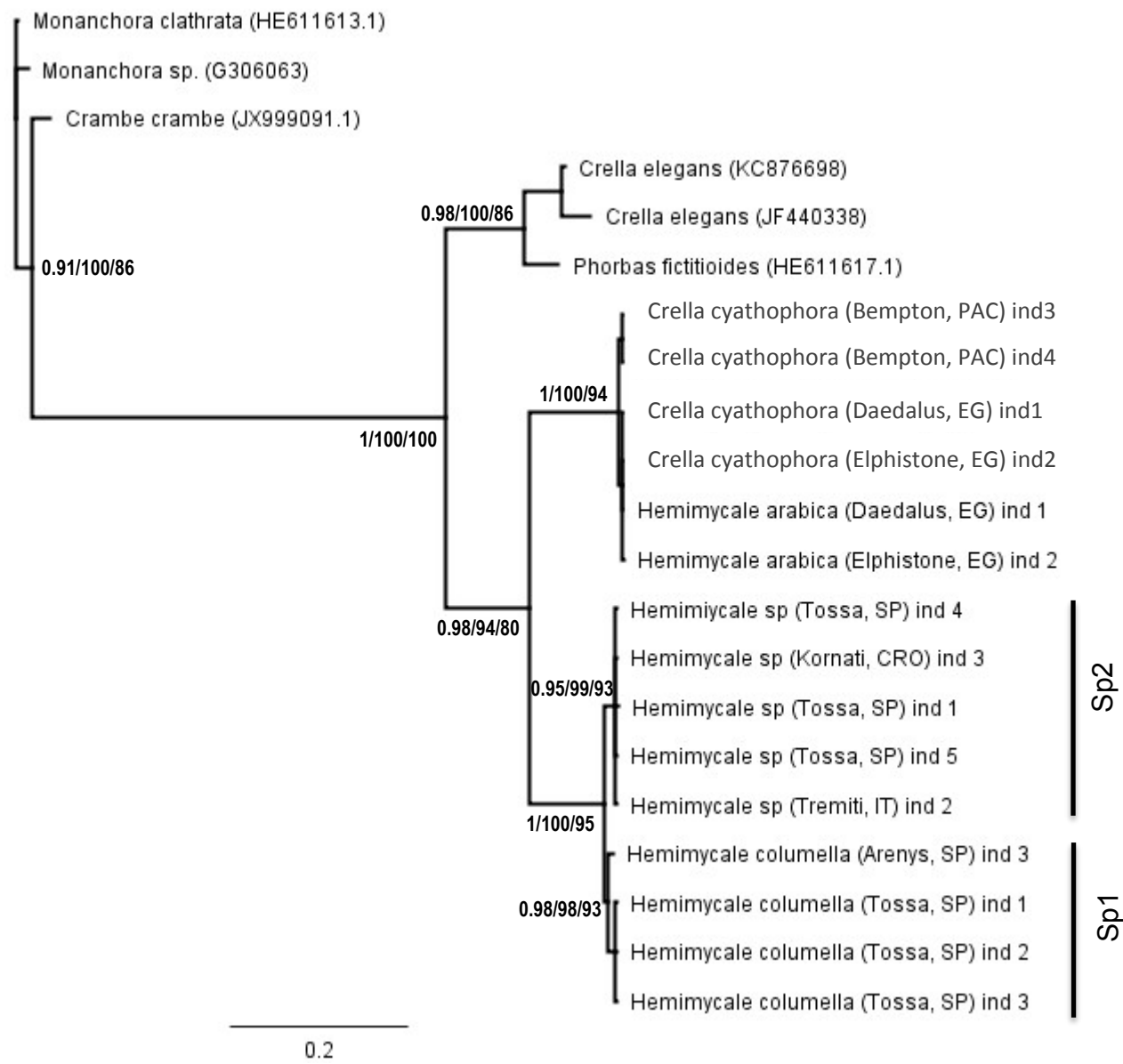

BI/NJ/ML

Supplement: Supplemental Information 4 — BI, NJ and ML gave the same topologies. Posterior probability, neighbor joining and maximum likelihood supporting values are at the base of clades. [file peerj-05-2958-s004.pdf]
